# Reduced representation of protein structure: implications on efficiency and scope of detection of structural similarity

**DOI:** 10.1186/1471-2105-11-155

**Published:** 2010-03-26

**Authors:** Zong Hong Zhang, Hwee Kuan Lee, Ivana Mihalek

**Affiliations:** 1 Bioinformatics Institute, A*STAR, 30 Biopolis Street, #07-01 Matrix, Singapore 138671

## Abstract

**Background:**

Computational comparison of two protein structures is the starting point of many methods that build on existing knowledge, such as structure modeling (including modeling of protein complexes and conformational changes), molecular replacement, or annotation by structural similarity. In a commonly used strategy, significant effort is invested in matching two sets of atoms. In a complementary approach, a global descriptor is assigned to the overall structure, thus losing track of the substructures within.

**Results:**

Using a small set of geometric features, we define a reduced representation of protein structure, together with an optimizing function for matching two representations, to provide a pre-filtering stage in a database search. We show that, in a straightforward implementation, the representation performs well in terms of resolution in the space of protein structures, and its ability to make new predictions.

**Conclusions:**

Perhaps unexpectedly, a substantial discriminating power already exists at the level of main features of protein structure, such as directions of secondary structural elements, possibly constrained by their sequential order. This can be used toward efficient comparison of protein (sub)structures, allowing for various degrees of conformational flexibility within the compared pair, which in turn can be used for modeling by homology of protein structure and dynamics.

## Background

The comparison of two protein structures is most efficiently handled as a hierarchical problem, more or less closely following the protocol laid out over a decade ago by Singh and Brutlag [1].

Its first step, fold recognition on the level of secondary structural element (SSE) correspondence, has been tackled repeatedly in the literature [2-14], building on the founding body of work related to aligning protein structures at the atomic-resolution level [15-19]. Most of the latter adopted an iterative resolution approach by starting from variously defined fragments of protein structure [20-23]. However, prominent methods capable of doing fast and conformationally tolerant search [4], such as SSM [12] and Fatcat [22] still take several tens of minutes of CPU time to perform a database search, doing a thorough but perhaps unnecessary job in order to eliminate bad candidates for a match. The development of complementary, ultra-fast methods for rigid structural comparison of proteins seems to have migrated to the realm of computer science, and typically relies on index or hash based database retrieval [24-29]. The algorithm from this family possibly the closest in spirit, if not in the scope, to the one we will propose below is TableauSearch[30]. With its high level of abstraction of protein structure, it indeed proves capable of searching databases approaching 10^5 ^entries as a matter of minutes. The method records and then discretizes the relative angle between any two pairs of SSEs in a structure, and stores it as a tableau [31] to be used in the database search. The entries, however, in TableauSearch database are rigid domains, and the algorithm thus dispenses with conformationally flexible searches right at the outset. Looking for a substructural match in this approach is not a completely straightforward affair either [30].

From mathematical biology side, several global descriptors of protein structure have been proposed [24] (for statistical takes on the problem see [32,33]) with an eye on organizing our knowledge of protein (or fold) universe [34]. An example is provided by SGM [24,35], a method that relies on a set of measures from differential geometry to describe overall structure of a protein domain, and which we will use below as a representative of its class of methods.

Even though the idea of a pre-filter based on gross features of the protein structure is implicit in several methods that have undergone a steady development [2,12], it has seldom been discussed and documented as a computational problem on its own. We propose, therefore, to take a look specifically at the question of the smallest possible set of features needed to describe protein structure, and propose its intuitively motivated reduced representation, equipped with a scoring function capable of detecting both rigid matches at domain level, and conformational changes involving relative motion of structural domains.

## Methods

In the current literature there exists a broad selection of methods for pairwise structural comparison of proteins [36-38]. Typically centered on backbone atom matching [18,21,39,40], they take several seconds or even several minutes to compare two structures and decide that the match is *not *possible. Why is it, then, that a human observer can establish, after a single glance that two protein structures in cartoon representation are (dis)similar? Certainly we are not mentally matching the backbone atoms, nor the angles through which the secondary structural elements (SSEs) are joined. Rather, the human observer will try to orient the two structures so that the SSEs point roughly in the same direction, and then perhaps check if the two diagrams are showing the same sequential ordering of the SSEs.

**Figure 1:**
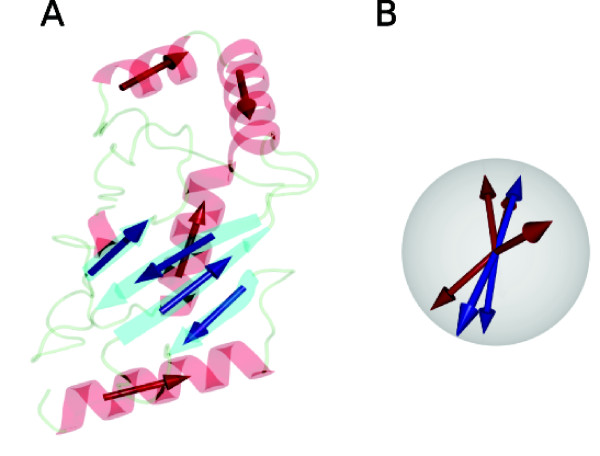
**Reducing the protein representation**. **(A) **Replacing the secondary structure elements (SSEs) by vectors retaining the information about the SSEs direction type (*α*-helix or *β*-sheet; indicated by red and blue colors). **(B) **Dispensing with the information about the SSE length and layout in space, leaves the representation consisting of unit vectors of two types, or, equivalently, points of two possible types on a unit sphere. The additional information missing from the illustration is the vectors' sequential order, carried over from the ordering of the SSEs on the protein sequence. All illustrations in this work were produced using PyMOL [DeLano, W.L. (2002) http://pymol.org] and POV-Ray [Persistence of vision raytracer (version 3.6, 2004) http://povray.org].

In search for an algorithm which will emulate this efficient process, we propose reducing the protein structure to the bare bones of structural information: SSE direction and type (Fig. 1) and sequential order. Furthermore, we define a function to score the match between two proteins in this representation; the best match between the two structures can then be found by looking for the rotation in the representation space which optimizes this function. The resulting algorithm enables searching through a database of protein structures in a way which is fast, independent of the size of SSEs and enables detection of structures which are related, but correspond to two different conformations.

### Structure representation and match scoring function

To reduce the size of the representation of the protein structure, we replace SSEs by direction vectors in space, while keeping the information about the SSE type (*α*-helix or *β*-strand), as indicated by the two different colors in the illustration in Fig. 1A. Furthermore, we drop the information about the elements' relative placement in space, resulting in the representation shown schematically in Fig. 1B. This representation can then be written as an ordered set of three-dimensional unit vectors,(1)

for a protein structure of *N*_*x *_SSEs. The order of the elements is determined by the order in which SSEs appear on the peptide sequence. Each vector represents one of the two types of structural elements that appear in protein structures: *α*-helix or *β*-strand. The information about the type is stored as a corresponding set of indicators

When this object is rotated, the rotation applies to each vector  equally: that is, the relative angles between the vectors stay the same. In this representation, the closeness of vector  from the representation of one protein structure, *X*, and vector , from the representation of the other structure, *Y*,

can be measured using the following matrix element, which falls off steeply as the angle between the two vectors increases:(2)

Here, *δ *is an adjustable parameter, and *R *is the rotation operator. The overall quality of the match is given by(3)

The negative sign here is arbitrary, indicating that *F*(*R*; *X*, *Y*) will be optimized through minimization with respect to rotation R. Choosing a small *δ *enables performing the double sum, while effectively summing only over the nearest matching pairs. In an ideal case of two exactly matching substructures, and *δ *tending to zero, the minimal value of *F *would be equal to the negative number of SSEs in the smaller structure. The distribution of values of *F*(*R*) in the rotation space will depend on the instance of the two sets, *X *and *Y*, under consideration. Depending on the orientation of vectors in *X *and *Y*, there may be a clear and isolated minimum in *F*(*R*), or there may be a region in *R *space densely populated with local minima of approximately equal depth. In particular, when comparing two structures with many nearly-parallel elements, covering a small solid angle in our reduced representation, a whole range of rotations *R *may conceivably result in false 'matches.' As an estimate of how easy it is to achieve a certain value of *F*, by a chance choice of rotation *R*, we evaluate the *z*-score:(4)

that is, the distance of the value *F *from the average over all rotations *R*, measured in units of standard deviation (the denominator in Eq. 4). The rotations resulting in near-zero (or even positive) *z*_*F *_can then be dismissed as insignificant given the geometry of the problem. The estimate of the *z*-score requires evaluation of the first two moments of *F*(*R*; *X*, *Y*) over the set of all possible rotations *R*, which can be done explicitly in the case of the average, and numerically in the case of the average square [Additional File 1].

To make possible a search through a database of realistic protein structures, we need a relaxed expectation of what is the best attainable match in the direction of SSEs [41], and therefore we need a finite sized *d*. This would lead to a number of false matches, were it not for the last requirement that we impose, which is that the sequential ordering of SSEs from the query is preserved in the set of their closest matching counterparts in the target. That is, we want the outcome of the algorithm to be a map *M*_*R *_: *i *↦*M*(*i*), where *i *is the index over  belonging to *X*, *M*(*i*) is the index over  belonging to *Y*, and for any two pairs (*i*_1_, *M*(*i*_1_)), (*i*_2_, *M*(*i*_2_)) it is true that(5)

where ≺ indicates the precedence on the primary sequence of the protein.

To find a map which preserves the order, we reinterpret *X *and *Y *as two alignable sequences of elements ("letters") labeled *i *and *j*. The letters here are SSEs, and their the similarity is given by *D*_*ij*_(*R*_*opt*_). *D*(*R*_*opt*_), Eq. 2, evaluated at *R*_*opt *_that optimizes *F*(*R*; *X*, *Y*) thus becomes a similarity matrix, playing the role that BLOSUM matrices, for example, play in the more familiar context of the alignment of two primary protein sequences. With the similarity matrix at hand, we can use a pairwise sequence alignment algorithm [42], such as Needleman-Wunsch or Smith-Waterman. The alignment procedure optimizes the sum of *D*_*ij*_(*R*) elements over the pairs (*i*, *M*(*i*)) matched in the pairwise matching algorithm(6)

Depending on the algorithm and parametrization, the alignment procedure may assign various gap penalties for the SSEs that do not map onto the other structure. *T *(*D*) ignores such SSEs. By retaining only the matched pairs which optimize *T *(*D*), we obtain a good orientation match between the pairs of SSEs, that at the same time complies with the sequential ordering in both structures.

A conformationally flexible match in this picture consists of two local minima in *F*(*R*; *X*, *Y*) for two different *R*'s, thus incorporating a model in which structural domains maintain their internal structural organization during conformational changes in a protein. In practice, it has to be verified that the two minima in *R *are different in a way that is statistically significant; the details may depend on the implementation.

Eq. 6 is the place to extend the formalism, if so desired, to include other quantities that can be ascribed to a pair of SSE, such as the length (mis)match between the two. In the numerical experiments we performed [Additional File 1], enforcing the length mismatch penalty of the form(7)

(with *ΔL*_*iM*(*i*) _the absolute value of the length difference, and *tol*_*ΔL *_an adjustable parameter) may improve the performance. The size of the improvement depends on the value used for *δ *and on the nature of the test the method is subjected to (i.e. do we count as a hit the cases of overall structural similarity allowing wide range of length mismatch in SSE, or not; how many levels of CATH classification we are trying to reproduce, etc.) [Additional File 1].

## Implementation

To determine whether the reduced representation of the protein structure contains enough information for a reliable database search, we have implemented the above ideas in a preliminary way. In this implementation we choose to optimize *F*(*R*; *X*, *Y*) starting from a set of initial guesses. This set consists of rotations constructed by choosing all possible combinations of two vectors from *X*, and two vectors of the corresponding type and same sequential order in *Y *; the initial rotation, then, is the one which puts the first two vectors from each pair in the same direction, and overlaps the planes spanned by each pair (see also Additional File 1). This is followed by the steepest descent search for a minimum in the space of quaternions representing *R *[43]. This loop is the central time consumer of the search. The number of pairs that need to be checked out is

where *s*_*i *_is the SSE type indicator, as in the previous section, and  here is the Kronecker *δ*, not to be confused with the Gaussian width parameter used in the rest of the paper. This leads to the worse case scenario (when all SSEs in both structure are of the same type) of complexity of , where *N*_*x *_and *N*_*y *_are the number of the SSEs in the two structures. This number may be substantially smaller (down to 0) in the case of mixed *α*/*β *structures. It can also be alleviated by grouping the directions nearly parallel in space, in which case the complexity becomes  where *n*_*x *_and *n*_*y *_are the numbers of distinct SSE directions in structures *X *and *Y*. (This compactification is later "unfolded" to do the pairwise alignment of SSEs.) In practice, the second and the fourth sum can be truncated at *j *= *i *+ *m *and *k *= *l *+ *m*, respectively, where *m *is a small number (2 or 3), without significant loss in performance. This makes the complexity *O*(*N*_*x*_*N*_*y*_), or *O*(*n*_*x*_*n*_*y*_) in the case of grouped directions.

In a pairwise comparison of two structures, the minima occurring at different *R*'s are stored if the *z *score is deemed statistically significant; later they are sorted in the order of the ascending *z*-score, and the best one is reported. A certain top number of suboptimal minima from this sorted list is checked for the complementarity of the match, and the complementary pair assigning the highest total score is reported as a flexible match. This part can be generalized to *n*, rather than only two complementary matches.

When doing a database scan, pairwise matches between the query and different target structures are scored and sorted using *T*(*D*) for a rigid search, Eq. 6, while the hits for a flexible search are sorted according to a heuristic score given by(8)

where *z*_1 _and *z*_2 _are the *z*-scores, Eq. 4, for *F*(*R*; *X*, *Y*) evaluated at two rotations, *R*_1 _and *R*_2 _which match two different structural domains, and *T *(*D*^max^), given in Eq. 6, is the quantity optimized in the pairwise alignment. The two maps corresponding to the two rotations result in two different matrices *D*_*ij*_(*R*_1_) and *D*_*ij*_(*R*_2_); *T *is evaluated based on the larger of the two values of matrix elements  = max [*D*_*ij*_(*R*_1_), *D*_*ij*_(*R*_2_)].

## Results

It is quite a strong claim that the three features we have selected (direction, type and sequential ordering of SSEs) provide enough information to distinguish two protein structures (and, conversely, to detect them as similar). While proving it may not be feasible, we may demonstrate that, statistically, it works quite reliably. Below we investigate the distinguishing and classifying power of the representation, and point out its two possible applications: annotation of novel structures and modeling of protein dynamics by homology. Parametrization, data sets, and related statistics can be found in Additional File 1.

### Self-scoring in a large database of structures

**Figure 2:**
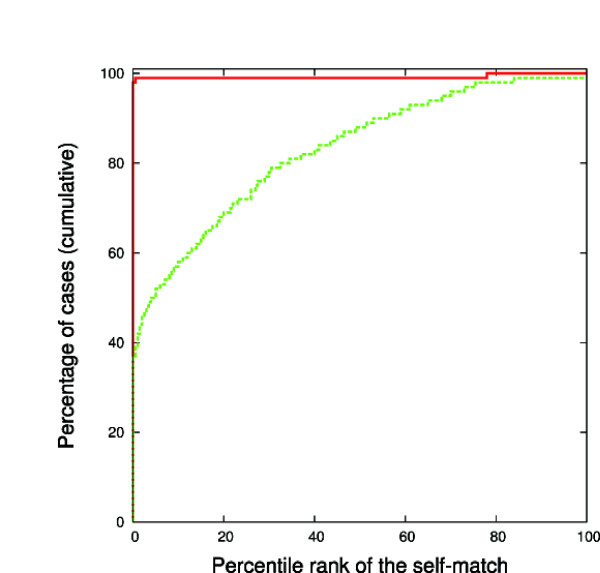
**Using the geometric criterion (Eq. 4) exclusively to detect the query in PDB25**. The histogram shows the cumulative percentage of cases for which the self-match score is found within the top percentage shown on the x-axis. Full line: using *δ *= 0.1, and dashed line: using *δ *= 0.3. For both choices of *δ *self match is always the highest on the list if the sequential ordering (Eq. 6) is used as an additional requirement (i.e. the graph looks the same as *δ *= 0.1 case shown in red here). Thus in a general comparison strategy one cannot rely on matching directions alone - additional constraints are needed to reduce the number of false positives.

As a necessary condition for the representation to be of practical value, it has to be unique, enabling the function F to find the query itself in a database of structures. To test this property, we ran an all against all search on 1000 structures from PDB25 [44] with more than three SSEs, using *δ *= 0.1 and *δ *= 0.3. For both choices of *δ*, the total aligned score, Eq. 6, is invariably highest for the self match. It is instructive to notice, though, that for the smaller value of *δ*, the geometric criterion, quantified by *z*_*F*_, Eq. 4, is in itself sufficient to distinguish uniquely the protein structure (full line in Fig. 2). However, as we allow for more fuzziness in the geometric match, by setting *δ *= 0.3, this criterion becomes insufficient (dashed line in Fig. 2), and we have to resort to the preservation of sequential ordering to describe the structure uniquely. This has direct implication on the following experiment, in which detecting structures which belong to the same fold but are not identical forces us to relax the demands on the exactness of the geometric match.

### Classification of structural domains

#### General performance characterization

**Figure 3:**
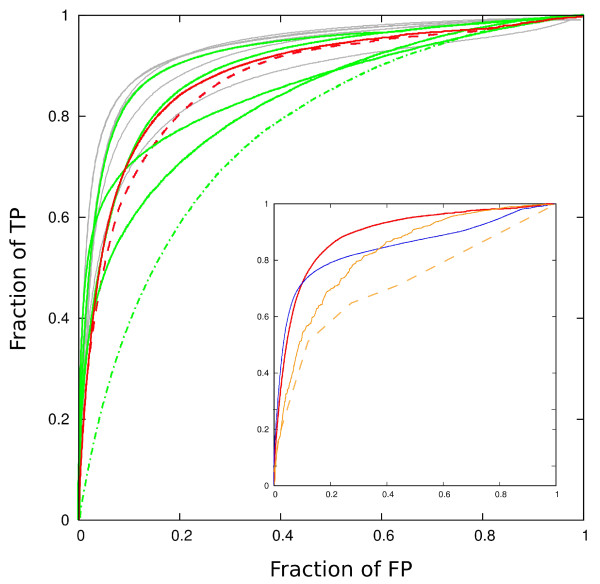
**Detecting structures from the same class, architecture and topology according to CATH classification**. To obtain an idea about the scope and resolution of the prefiltering we are proposing (red), the results are shown on the same graph with representative full resolutions methods. It should be understood here that the prefiltering step we are proposing is some 40 to 1000 times faster than the full resolution methods. (The purpose of full resolution methods, of course, is achieving the high quality of the alignment, rather than the speed database scanning. The quality of pairwise alignment is not tested in this type of experiment.) The results are presented in terms of a ROC curve: for a sliding threshold in the quality score, the number of true positives (TP) above the threshold (y-axis) is shown as function of the fraction of false positives (FP) falling above the threshold (x-axis). Red line: the ROC curve using the total aligned score (Eq. 6) to rank the quality of the match, with *δ *= 0.5 (full red line) and with *δ *= 0.3 (dashed red line) and gap opening penalty of -1 in the alignment step. Gray: various high resolution methods (CE, [21]; STRUCTAL, [16]; LSQMAN, [58]; DALI, [17]; SSM, [12]; SSAP, [15]) scored using using SAS score [16]. For the original context, timings, and discussion see Kolodny *et al*. [38]. Green line: "generation 2000" high resolution methods, in the order of decreasing area under the ROC curve (and, roughly, the time taken for the task): 3Dhit [9](80 CPU hrs), TMalign [13](80 CPU hrs), SABERTOOTH [14](60 CPU hrs), MAMMOTH [8](40 CPU hrs). Green, dash-dotted: SGM [24] (several CPU minutes). Inset: comparison of the method discussed in the text with the pre-filters used in VAST and SSM, on a smaller data set, acceptable to all three methods [Additional File 1]. Red line (full): the ROC curve using the total aligned score (Eq. 6); blue line (full) pre-filter used in VAST; orange line (full): SSM pre-filter optimized for performance on this type of a test; orange line (dashed): native SSM pre-filter.

To be of use, a method of the type we are presenting is expected to be able not only to find and rank highly all structures which are identical to the query, but also the structures which are, in some sense, similar to it. Using the test (and the test set, reduced by the number of structures with less than four SSEs) proposed by Kolodny *et al*. [38] we performed an all-against-all comparison of over 2000 structures from CATH v.2.4 [45], and measured the ability of the method to rank highly the pairs with the same class, architecture, and topology. The outcome is shown in Fig. 3, in the form of receiver operating characteristic (ROC) curve. The best methods climb steeply in the fraction of true positives for small values of false positives. To place the performance of our approach in the context of the existing methods, we show the results for several high-resolution methods (data in gray originally collected and discussed by Kolodny *et al*., [38]; green: data additionally collected in this work [8,9,13,14]), and one low resolution (SGM, [24]; green) method on the same graph. In all computational experiments in this paper the methods were considered in their pairwise mode - additional capabilities of the accompanying servers (such as hierarchical clustering in the target database, hash-based target retrieval etc) were not the subject of investigation. The method suggested here lies within the bounds of performance characteristics of methods with finer granularity (that is, methods that perform the actual alignment between two structures), indicating that direction, type, and order of SSEs represent a sizeable portion of the signal that the detailed methods are picking.

Consistent with the qualitative description underlying the CATH classification, in this experiment we obtained better sensitivity/specificity tradeoff by relaxing the criterion for the directional match by setting *δ *= 0.5. Together with the results for the exact match problem above, this suggests a possibility of simulated annealing approach with stepwise decreasing *δ*, to obtain a distribution of hits with varying strictness in structural similarity to the query. (For a remote structural similarity, no significant value of *F *may be obtainable for very small *δ*. Conversely, large *F *for small *δ *indicates a very small variation in direction of SSEs, typically related to strong overall similarity.)

The time taken for this computational experiment, of approximately 170 CPU minutes for *δ *= 0.5 (and 90 minutes for *δ *= 0.3) on a 3 GHZ processor, compares favorably with some 40 to 1000 CPU hours needed for the high resolution methods to complete the same task (see Fig. 3 and experiments in [14,19,46]). It should be kept in mind, of course, that the approach we have laid out functions only as a pre-filter and classifier, and does not produce the actual structural alignment on the atomic level. Rather, we argue, a small subset of the structural features is responsible for the classification we are trying to reproduce, and this can be used to economize with computational resources.

#### Comparison with pre-filters used in other methods

Among the existing tools for structure comparison, the most proficient ones do include some way of pre-filtering (e.g. the difference in sum of rows and columns in the contact matrix used by DALI, [40]), discussed to a larger or smaller extent. Since most of such methods have been designed to pass the test like the domain classification discussed here, this is a reasonable place to point out the novel aspects of our reasoning, and their performance implications. Hence we conclude this section with a brief comparison with VAST and SSM, both well established methods with their own servers [2,12,47], and both using SSEs as the basic elements of protein structure.

#### SSM

In the course of development of SSM, the authors have devoted a whole publication [48] to the discussion of its preliminary step. In its pre-filtering stage, SSM constructs a graph for each structure. The vertices of the graph carry the information about each SSE (length, type), while the edges are associated with the attribute of each pair of vertices/SSEs they are connecting (distance, angles). The algorithm then looks for a common subgraph using a set of heuristic rules, divided into 5 levels, to decide on the "sameness" between vertices and edges from the two graphs. The necessity to introduce the cutoffs (in order to deduce the existence of common subgraph elements) enforces discretization of the scoring function, in contrast to the approach proposed in this work. An attempt to run the CATH experiment using the five discrete levels of similarity in SSM is shown in Fig. 3, dotted line in the inset.

Despite the comparatively small area under the ROC curve obtained this way, the information manipulated by SSM is actually quite seizable. In an attempt to extract more resolution from the SSM pre-filtering, we took the outcome from all five levels simultaneously, and treated the optimization of the ROC area as a machine learning problem [Additional File 1]. The outcome is shown in Fig. 3, inset, blue line. It approaches the ROC achieved by the method we are proposing, but at the cost of introducing fifty parameters difficult to grasp intuitively.

#### VAST

VAST also relies on a graph matching tactics in its pre-filtering stage, but in a substantially different way ([2,47]; also see [49]). In contrast to SSM, where one graph represents each structure, in VAST graph is constructed for a *map *between two structures, as follows.

In VAST, SSE elements are represented by direction vectors that, aside from the information about the type, explicitly retain the information about the SSE length (i.e. while our representation consists of unit vectors, VAST uses vectors of the length proportional to the length of the SSE). A discrete set of rotations acting on one of the structures/representations is attempted, each rotation satisfying the following: (i) the rotation maps one of the vectors from the rotated structure exactly onto one of the vectors from the fixed structure (defined to be the *z *direction), and (ii) it brings another vector from the rotated structure into the plane defined by *z *and some vector from the fixed structure. This contrasts with the continuous space of rotations our algorithm is exploring. In both (i) and (ii) the vectors mapped between the two structures have to be of the same type.

For each of the rotations from this set, a structure termed "digraph" is constructed: the vertices correspond to *pairs *of vectors, one from each structure, that fall within certain cutoffs regarding the angle between the two and the allowed distance between their endpoints. Digraph's edges exist if the two pairings involved respect the sequential ordering on the input structures, and if they carry the weight inversely proportional to the difference in the angles between two vectors corresponding to the same structure. The cutoffs and the weight are parametrized and the parameters optimized for a typical search. In our comparison runs we used the default set of parameters. The digraph can be traversed quickly to find the best alignment for a given rotation, and consequently the best scoring alignment over all rotations chosen.

This algorithm is similar to certain extent to the one proposed in this work, in the limit of small Gaussian width *δ *(which does not allow for much exploration of rotational space away from the initial guess), and with the length penalty (Eq. 7) included. The digraph traversal is an alternative to the dynamic programming approach we are taking.

The crucial difference here, however, is that VAST operates on the level of *pairs *of SSEs, somewhat analogous to the possibility of using *F*^2 ^as a scoring function in our approach. The requirements this imposes are stricter, explaining the somewhat higher sensitivity of VAST in the low false positive region (Fig. 3; it might be worth re-iterating here that in considering Fig. 3 one should keep in mind that the method in question is not VAST proper, but, rather, its pre-filtering stage only.) The rotations, furthermore, that are tested are not optimized to match the rest of the structure (only the pair of pairs defining the rotation) and thus are not readily applicable to further refinement of the matching transformation, an extension that our approach is in principle amenable to. Also, insisting on the length match (something that we do not do necessarily), in order to get rid of false positive matches, is a strategy which might backfire in the attempts to extend the search to a more distant structural similarity.

As is, however, this method of pre-filtering is very fast (the initial guess for a rotation is not further optimized, a major time consuming step in our approach), and slightly better in the small FP region than our best take on the CATH test, (Fig. 3; inset; full blue line). Nevertheless, our ROC does show signs of greater robustness and higher resolution capability, as indicated by the larger area under the curve.

### Detecting a substructure in a set of larger structures

**Figure 4:**
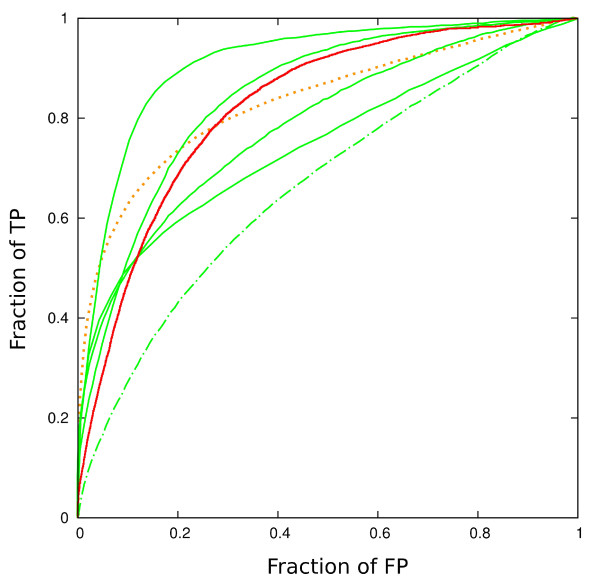
**ROC for finding a domain in the full protein structure**. Full red line: the method discussed in the text, with *δ *= 0.5 (15 CPU min). Dotted orange line: SSM [12](several tens of CPU), dash-dot, green: SGM ([24], Full green line: "generation 2000" high resolution methods, in the same order of appearance as in Fig. 3 (by descending size of the area under the ROC curve): 3dhit [9](6 CPU hrs), TMalign [13](8 CPU hrs), SABERTOOTH [14](5.5 CPU hrs), MAMMOTH [8](3.5 CPU hrs). These methods, as well as SSM, do the high resolution alignment, which both makes them more reliable in the high specificity (small FP) region of the graph, and slows them down.

What is the resolution with which this approach can detect a smaller query structure within a larger target structure? To obtain an estimate we took the test set from the previous experiment (see "Classification of structural domains" subsection) and required for each domain to be matched to the target domain with the same CAT number from CATH classification, but this time with the target domain embedded in the original full-protein structure. The results are shown in Fig. 4. Again, we try to place the performance of this rough comparison method in the context of what is achievable by methods performing the full structural alignment. In this we choose to stick with the methods that are fast enough to perform the test in a reasonably short time. For their performance in comparison with slower full-alignment methods see Fig. 3.

To indicate an *im*possibility first, methods which assign a set of global descriptors to the structure (such as SGM, dash-dotted line in Fig. 4) cannot tackle this problem. SGM is used as an example here: this is equally true for all methods relying on assigning a hash function to a predefined structural domain.

In the following, we choose not to reinterpret the methods used, and refer the reader to the original publications. Instead, we would like to highlight the specific algorithmic choices, which, we believe, give these methods advantage over other approaches, in particular the one proposed in this work. Notably here, MAMMOTH [8] and SABERTOOTH [14] were never designed with large database scanning primarily in mind (even though they perform well in that respect), but rather with the goal of comparing a model structure to a template (MAMMOTH) or obtaining a precise structural alignment between two structures (SABERTOOTH).

The basic unit of structure being compared in MAMMOTH [8] is a heptapeptide. Compared to our current implementation, which enforces an SSE as an elementary unit, irrespective of its length (see "Discussion"), the heptapeptide approach enables better resolution in the high specificity region.

Sabertooth [14] uses the idea that the correct alignment may be, among other things, recognized by the similar environment ("connectivity pattern") seen by the aligned structural motifs. This approach also results in strong performance in the small FP regime, and something that our approach hardly generalizes to.

TMalign [13] uses SSE alignment, before trying SSE mapping which would be a nonsense from the sequence point of view. This is an opportunity our implementation misses (see the the first paragraph in "Implementation" section). Otherwise, TMalign manipulates very similar input information to the approach proposed here, and is therefore encouraging to see it trace out a practically identical ROC curve, both in this test (Fig. 4), and in the test shown in Fig. 3.

3Dhit [9] shows the most robust ROC curve in this test. It dissects the peptide into 13-residue fragments, which first conveys the same advantage over our approach as discussed for MAMMOTH above. The algorithm then chops up the fragment into clusters. In mapping clusters the identity of amino acid (types, presumably) is enforced [50], an algorithmic move that speeds up the search substantially, but we would like to stay away from as long as possible in development of our algorithm. Further on, however, the fact is exploited that for similar structures a transformation that matches two subsets will match larger regions of protein structure, the fact that our approach also builds on, and that can be used to increase resolution of the match toward a full backbone match.

SSM [12], a veteran high resolution method, does the best in the small FP region, but at the cost of CPU time two orders of magnitude longer than required to do the rough comparison we are proposing here. The high resolution is achieved by imposing a series of geometric requirements on matched *pairs *of SSE from the two structures (see the sections "Comparison with pre-filters used in other methods," and "Discussion," as well as the original publication, of course).

The overall message seems to be that judging by the speed of our prefiltering and quite competitive sensitivity/specificity tradeoff it achieves it provides a good base for protein comparison engine.

### Finding conformationally related structures

**Figure 5:**
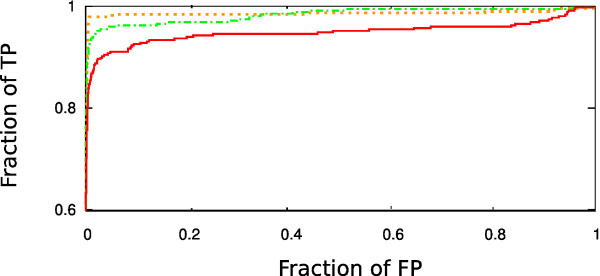
**Ranking of the conformationally related partner, with the same primary sequence, for 361 queries in "Dyndom" experiment**. Dotted orange line: SSM [12], dash-dot, green: SGM [24], full, red: the method discussed in the text, with *δ *= 0.3 and length mismatch penalty.

**Figure 6:**
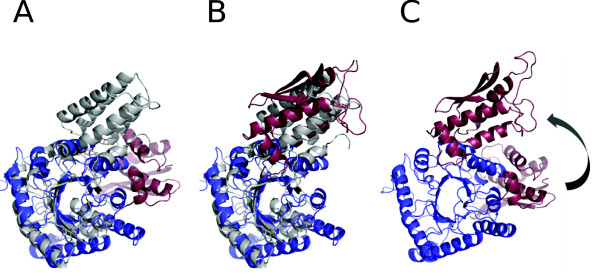
**A conformational transformation candidate, as modeled by structural matching**. Enolase (PDB [59] identifier 1pdy; white) and signal recognition 54 kda protein (1j8yF; N-terminal red, C-terminal blue). Hypothetical use of enolase as the scaffolding to model conformational change in the signal recognition particle. **(A) **Applying the linear coordinate transformation which overlaps the larger (C-terminal) domain of the two proteins to both domains in the signal recognition particle. **(B)** Transforming both the larger and the smaller domain separately to obtain the maximal overlap with enolase structure. **(C) **The model for conformational change in the signal recognition particle, based on (A) and (B). Note that the structural match in itself does not in itself imply functional relatedness nor common evolutionary descent.

Next, we need to establish that the representation allows detection of conformationally related structures. As an easier sub-problem, we first consider the task of finding the same protein in two different conformations, thus taking the noise brought in by the evolutionary divergence out of the equation. This is analogous to the first test ("Self-scoring in a large database of structures") in that the primary sequences of the query and the target are the same, but this time their structures correspond to two different conformations. The test set consists of 677 structures selected from the database created by the users of Dyndom [51,52] server. A similar set of of pairs of conformations of the same chain could have, of course, been created from other sources, for example MolMovDB [53]. The results are shown in Fig. 5, together with the performance on the same test for one slow, high resolution (SSM, [12]) and one fast, low resolution method (SGM, [24]). The reduced representation shows reasonably high resolution, even though for this particular problem there are better suited approaches. SSM and SGM, for instance, both perform exceptionally well. In particular, the total time requirement for SGM is several minutes to produce the results shown. (SSM, being a high resolution method, takes several CPU days.) The harder problem in the same category is finding conformationally related pairs of protein structures, distantly related (or unrelated) in the primary sequence. This problem is compounded by the problem of finding the test set itself (finding such pairs actually being one of the original motivations for the algorithm development). To illustrate an application for a computational tool which can successfully handle such cases, we briefly comment on an example marked as a flexible match in an all-against-all experiment on PDB25. Fig. 6 shows a structural match between enolase and signal recognition 54 kDa protein. Signal recognition protein bears in its C-terminal apparently so far undescribed similarity to Pfam [54] N-terminal enolase domain. The smaller domain in this protein also bears some similarity to the C-terminal enolase domain, at least in its *α*-helical part. The four domains, however, have been classified in CATH as 3.30.390.10 and 3.20.20.120 (enolase N- and C-terminal domains respectively) and 1.20.120.140 and 3.40.50.300 (signal recognition particle, N- and C-terminal domains). Taking into account no more than 8% in sequence identity in the aligned regions in both domains, it means that this example would be difficult to construct either by a search though homologous sequence space or by inspection of CATH database. The aligned core domain in C-terminal domains, however, shows striking structural similarity (see the related movie in Additional Documentation [Additional File 2]).

The larger and the smaller domain appear rearranged with respect to each other in the two structures, as can be seen by using a linear coordinate transformation that overlaps the larger domain (Fig. 6). One structure, this suggests, could then be used as a scaffolding to model conformational change of the other. Could this conformational change appear as part of physiological functioning of either of the two proteins? Is it at all physically possible to get from one conformation to another at room temperature? Finding examples with structural similarity in two structural domains, rearranged in space, may indicate a possibility of conformational change in one or both proteins. The reasonableness of such conjecture is, of course, subject to further testing through targeted molecular dynamics, or some related approach. The type of structural comparison we are advocating, however, should produce more examples for this type of study.

## Discussion

We have suggested in the Methods section that after dispensing with translation and the length of the SSEs, the structure is effectively represented by a set of points on a unit sphere. With these points we associated information about underlying SSE type and sequential order. By settling on the minimal representation of the protein structure, we set out to analyze its sufficiency for structure description and retrieval.

As noted in subsection "Self-scoring in a large database of structures", Fig. 2 and the related discussion, the directions themselves, except when taken very narrowly (*δ *= 0.1 in our formulation), may be matched by quite diverse protein structures. To get rid of false positive matches that arise that way, we have suggested imposing the requirement that the matched SSEs follow the same sequential order in the two structures. This, however is not the only possible way around the problem: as discussed in Mizuguchi and Go [5], and later elaborated by Krissinel and Henrick [12] in development of SSM (discussed above), the directions of SSEs can be supplemented by various other pieces of information: the length of SSEs, the distance and torsion angle between all possible *pairs *of SSEs in a structure and/or the angles between their directions and the direction of the line passing through their geometric centers. The advantage of using this type of information, rather than requiring the common sequential order of the SSEs, then is in the ability to look for pairs of proteins with different connectivity between SSEs, that still result in the overall comparable structures. On the flip-side, the set of requirements might end up being too restrictive in the search of similar (but non-identical) structures, as we have illustrated in the inset of Fig. 4.

Contrary to the model of similarity adopted here, where similar structures are assumed to share to certain extent the underlying SSE arrangement, it is conceivable that two proteins might share a common function as long as they offer a common geometry of the surface to their common (or similar) interacting partners [55]. In that case one might be interested in a method for detection and retrieval of proteins sharing the same shape, irrespective of the underlying secondary structure. It is a possibility not explored here. Methods for retrieval by global shape similarity have been discussed in literature (see [29] and references therein) and extremely short retrieval times (~10^-4^*s *on a 3 GHz CPU) reported [29]). Some questions remain outside the scope of these methods, such as detection of a common substructure or structural motif.

Sticking to a more conservative model of shared protein structure, the problem which ultimately needs to be resolved is the correspondence: which SSEs (and later, on a finer detail level, which backbone atoms) on two structures correspond to one another. Function *F*(*R*; *X*, *Y*) enables us to initially sidestep this problem, in principle at least, because the fast fall-off of the closeness measure *D*_*ij*_(*R*) (Eq. 2) makes possible the double sum over all elements without the danger of obtaining as the optimal a solution where no actual match exists, but the sum over many distant neighbors artificially increases the score. By starting the protein structure comparison by minimization of *F*(*R*; *X*, *Y*), we are effectively adopting, on the SSE level, the match-first-align-later approach, popularized by Gerstein and Levitt [18] (see also [56] for a further development of the idea).

Ideally, the scoring function *F *would quantify, in a single expression, the geometric match under the constraint of sequential ordering of the pairs, a problem which we leave open. On the high-resolution end of the spectrum of related ideas lies the URMS-RMS hybrid algorithm [8,23,57]. There, a set of directions in space is also considered, however not along the SSEs, but along the lines connecting neighboring *C*_*α *_atoms within a heptapeptide. Being a high-resolution method, it comes with the computational burden comparable to the other backbone-matching approaches (and, of course, with the final reward of the actual detailed matching of two backbone traces). The match scoring function used in that work is different from the one suggested here, but it runs into a similar difficulty of estimating the statistical significance for a match of different structures. A solution offered there is comparison with an empirically derived background distribution of match probabilities using existing, unrelated protein structures.

Instead, we opted for a solution which separates the geometric match from the alignment. The fuzziest point in the algorithm we have outlined, therefore, is that the averages in Eq. 4 should properly be evaluated not over the set of all rotations *R*, but only over those rotations which allow, through the matrix *D*_*ij*_(*R*), the alignment of subsequences of the two proteins of substantial length. Numerical evaluation of these proper averages would effectively grind the search to a halt, so in our prototype evaluation we keep the averages over all *R *as an approximation. The approximation works well for the rigid search, where it is used to dispense with bad solutions, rather than score good ones. In the case of the flexible search we resort to the total assigned score as a scoring function, coupled with the requirement that both maps have a high rotational *z*-score on their own.

In terms of the implementation, the room for improvement is certainly ample. The relatively large number of false positives is attributable, at least in part, to parallel beta sheets and helix bundles, which can be amended by more careful grouping of the representation vectors. Also, in the implementation used here, each *β *strand is represented by a single vector  - a rather crude approximation for most *β *strands, which are often bent.

Perhaps stating the obvious, the ultimate degree of success of an approach will depend on the choices made in the implementation, as much so as on the underlying idea. In this work, the available implementations (steepest descent and Needleman-Wunsch) decided the way in which the three features we selected to describe a protein were used. Even though a faster, or more robust, implementation could perhaps be achieved by a different choice of optimization or alignment algorithm, these are replaceable components, and the main points of improvement are in the representation itself, in the distance (or match scoring) function, and in its statistical evaluation.

## Conclusions

In an attempt to build a pre-filtering tool for a search through a database of protein structures, we proposed (i) reducing the representation of protein structure to an ordered set of unit vectors carrying the information about the direction and the type of the secondary structure element they represent, Eq. 1, (ii) measuring the distance between two elements of the same type in terms of a quantity falling off exponentially with the increasing angle between the two, Eq. 2, (iii) measuring the distance between the two representations as sum over pairwise distances between their elements, Eq. 3, and (iv) ordering the near matches by their total aligned score, Eq. 6.

The representation is easily extendable to other descriptors of protein geometry by generalization of the type, currently restricted to *α*-helix or *β*-strand, and interesting statistics may result from allowing the Gaussian width *δ *to be type dependent.

We have shown that an implementation which minimizes the distance defined in Eq. 3 through a steepest descent calculation, and subsequently enforces the sequential order between the matched SSEs using standard sequence alignment approach, performs well in terms of the resolution in the structure space. Notable, also, is the speed that can be achieved in structure comparison without tying up the information in the form of a single index - it is precisely this feature which enables us to generalize the search to flexible and multidomain cases, and makes this idea uniquely versatile among structural comparison algorithms. The main concern addressed in this work has been whether this minimalist description of protein structure contains enough information to uniquely describe protein (sub)structures, and structural classes. The conclusion is that the information is certainly sufficient for a unique self match of each protein structure studied (Fig. 2), and represents the large chunk of the signal detected by the high resolution methods.

(Fig. 3). When extended to detection of distant structural similarity, the approach starts to suffer from "false positive" matches (note that the information about the translational degrees of freedom is absent), but it stays within the acceptable limits of accuracy set by high resolution methods, and its speed certainly allows for an improvement by extending the number of elements and types in the description.

The straightforward motivation for this description of protein structure makes clear what the pitfalls and directions of improvement are, but even the existing implementation indicates that the approach may prove valuable in making novel predictions, in terms of both rigid and flexible structural comparison. The server to accompany this paper, as well as the code used in the analysis presented in the text is available a http://epsf.bmad.bii.a-star.edu.sg.

## Abbreviations

SSE: secondary structural element; ROC: receiver operating characteristic; FP: false positive; TP: true positive.

## Authors' contributions

ZHZ contributed to the code development and data analysis and presentation; HKL and IM developed the algorithms and drafted the manuscript; IM designed the study and the code implementation. All authors read and approved the final manuscript.

## Supplementary Material

Additional file 1 **Additional Documentation**. A pdf document, containing details of algebraic manipulations, and description of data sets and parametrization. Click here for file

Additional file 2 **core region.mov**. A short movie in mov format, showing the overlap of core regions of of larger domains of enolase and signal recognition 54 kDa protein, discussed in "Finding conformationally related structures" section. Click here for file
