# Supplementary material for: Reduced representation of protein structure: implications on efficiency and scope of detection of structural similarity
Source: BMC Bioinformatics. 2010 Mar 26;11:155. doi: 10.1186/1471-2105-11-155 (PMC3098053; doi:10.1186/1471-2105-11-155)
Supplement: Additional file 1 — Additional Documentation. A pdf document, containing details of algebraic manipulations, and description of data sets and parametrization. [file 1471-2105-11-155-S1.PDF]

Reduced representation of protein structure: implications on efficiency and scope of detection of structural similarity - additional documentation

Zong Hong Zhang, Hwee Kuan Lee and Ivana Mihalek

January 26, 2010

Average and average square of the scoring function F.

To estimate the z score of the value of function F for a given rotation

$$F(R;X,Y) = -\sum_{i=1}^{N_x}\sum_{j=1}^{N_y} s_i s_j e^{-|\vec{y}_j - R\vec{x}_i|^2/\delta^2} \tag{1}$$

we need to know its average and average square over all rotations. The average of F over all rotations, $\langle F\rangle$ is defined as

$$\begin{aligned}\langle F(R;X,Y)\rangle &= -\sum_{i=1}^{N_x}\sum_{j=1}^{N_y} s_i s_j \langle e^{-|\vec{y}_j - R\vec{x}_i|^2/\delta^2}\rangle \qquad (2)\\ &= -\sum_{i=1}^{N_x}\sum_{j=1}^{N_y} s_i s_j \frac{1}{8\pi^2}\int_0^{2\pi} d\phi \int_0^{2\pi} d\psi \int_0^{\pi} sin\theta d\theta e^{-|\vec{y}_j - R(\phi,\theta,\psi)\vec{x}_i|^2/\delta^2},\end{aligned}$$

using the "x-convention" for Euler angles ϕ, θ, and ψ. The integral can be evaluated for every pair $(\vec{y}_j, \vec{x}_i)$ by orienting the coordinate system so that initially $\vec{y}_j$ and $\vec{x}_i$ point in $\hat{z}$ direction. (What we pick as an initial orientation is immaterial, since we are going integrate over all orientations anyway). Any rotation $R(\phi,\theta,\psi)$ in that system results in $\vec{x}_i \cdot \vec{y}_j = cos\theta$, irrespective of the values of ϕ and ψ, as illustrated in Fig 1. (This integral, involving only one vector from X and Y sets, is independent of relative orientations of vectors within each set, in contrast to the integrals appearing in the evaluation of $\langle F^2\rangle$, see below). The integral then evaluates straightforwardly:

$$\begin{aligned}\langle F(R;X,Y)\rangle &= -\sum_{i=1}^{N_x}\sum_{j=1}^{N_y} s_i s_j \frac{e^{-2/\delta^2}}{2}\int_0^{\pi} sin\theta d\theta e^{2cos\theta/\delta^2} \qquad (3)\\ &= -\frac{\delta^2}{4}\left(1-e^{-4/\delta^2}\right)\sum_{i=1}^{N_x}\sum_{j=1}^{N_y} s_i s_j \\ &= -\frac{\delta^2}{4}\left(1-e^{-4/\delta^2}\right)\left(N_x^H N_y^H + N_x^S N_y^S - N_x^H N_y^S - N_x^S N_y^H\right).\end{aligned}$$

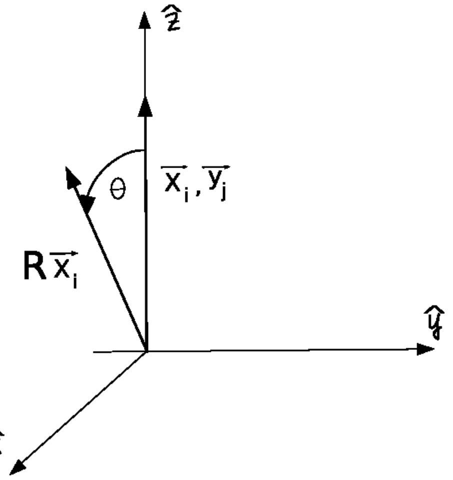

Figure 1: The coordinate system orientation used in the evaluation of the integral in Eq. 2.

In the last line of the Eq. 3 we note that in this simple case even the sum over the values of the indicator function S (Eq. 2 in the main text) can be expressed in terms of the number of helices, N^H, and strands, N^S, in the two sets.

In the case of F^2, needed for the evaluation of the standard deviation, which in turn provides the yardstick for the distance from the average of an evaluated F (see the main text, discussion of the z score), we resort to quaternions [1] to be able to handle the calculation in a systematic way:

$$\begin{aligned} \langle F^2 \rangle &= \sum_{i=1}^{N_x} \sum_{j=1}^{N_y} \sum_{k=1}^{N_x} \sum_{l=1}^{N_y} s_i s_j s_k s_l \langle e^{-|\vec{y}_j - R\vec{x}_i|^2/\delta^2} e^{-|\vec{y}_l - R(q)\vec{x}_k|^2/\delta^2} \rangle \\ &= \sum_{i,k=1}^{N_x} \sum_{j,l=1}^{N_y} s_i s_j s_k s_l \langle e^{-|[0,\vec{y}_j] - q[0,\vec{x}_i]\bar{q}|^2/\delta^2} e^{-|[0,\vec{y}_l] - q[0,\vec{x}_k]\bar{q}|^2/\delta^2} \rangle. \end{aligned} \tag{4}$$

Conveniently for our purposes here, it can be shown [1] that

$$|[0, \vec{y}_j] - q[0, \vec{x}_i]\bar{q}|^2 = q^T A^T(\vec{y}_j, \vec{x}_i) A(\vec{y}_j, \vec{x}_i) q, \tag{5}$$

where $A(\vec{y}_j, \vec{x}_i)$ is given by

$$A(\vec{y}_j, \vec{x}_i) \equiv \begin{pmatrix} 0 & -b_1 & -b_2 & -b_3 \\ b_1 & 0 & -a_3 & a_2 \\ b_2 & a_3 & 0 & -a_1 \\ b_3 & -a_2 & a_1 & 0 \end{pmatrix}, \begin{cases} \vec{a} \equiv \vec{y}_j + \vec{x}_i \\ \vec{b} \equiv \vec{y}_j - \vec{x}_i \end{cases}.$$

The average in the last line of Eq. (4) thus becomes

$$\begin{aligned} \langle e^{-|[0,\vec{y}_j] - q[0,\vec{x}_i]\bar{q}|^2/\delta^2} e^{-|[0,\vec{y}_l] - q[0,\vec{x}_k]\bar{q}|^2/\delta^2} \rangle &= \langle e^{-q^T \Sigma q/\delta^2} \rangle, \\ \Sigma \equiv A^T(\vec{y}_j, \vec{x}_i) A(\vec{y}_j, \vec{x}_i) + A^T(\vec{y}_l, \vec{x}_k) A(\vec{y}_l, \vec{x}_k). \end{aligned}$$

Parameterizing the quaternions with 3 angles κ, θ, and ϕ (to maintain the normalization to 1)

$$q = [\cos\kappa, \sin\kappa\sin\theta\cos\phi, \sin\kappa\sin\theta\sin\phi, \sin\kappa\cos\theta],$$

we obtain

$$\langle F^2 \rangle = \sum_{i,k=1}^{N_x} \sum_{j,l=1}^{N_y} s_i s_j s_k s_l \frac{1}{2\pi^2} \int_0^{\pi} \sin^2\kappa d\kappa \int_0^{\pi} \sin\theta d\theta \int_0^{2\pi} d\phi e^{-q^T(\kappa,\theta,\phi)\Sigma q(\kappa,\theta,\phi)/\delta^2} \quad (6)$$

In the current implementation of the method, the values of this integral are tabulated for discretized values of the angles $\angle(\vec{x}_i, \vec{x}_k)$ and $\angle(\vec{y}_j, \vec{y}_l)$.

It is interesting to note that this way all moments of function F can be evaluated at practically the same computational cost. Among other, the first moment, available explicitly (Eq. (3)) can be used to check the numerical implementation.

Data sets

When testing the ability of the representation to find itself in a database ("Self-scoring in a large database of structures"), the test set consisted of 1000 structures from PDB25[2] [ftp:/ftp.embl-heidelberg.de/pub/databases/protein_extras/pdb_select], a database of structures with not more then 25% identity in their primary sequence. Broken down to the CATH-classifiable domains, the set contained almost exactly one quarter (25%) mainly α, one quarter (24%) mainly β, and one half (51%) mixed α and β classes, covering 70 different architectures. In all experiments we required that the structure consists of at least 4 SSEs, and we relied on the PDB[3] header for the information about the secondary structure.

In the first CATH-related experiment (to be referred to as "CATH experiment"), matching pairs of domains (see subsection "Classification of structural domains" in the main text), we borrowed the selection made by Kolodny *et al.* [4] in their publication. This selection reduced by the set of domains with less than 4 SSEs or coming from a PDB entry with problematic header, resulted in a set of 2132 structure. Out of $2132(2132-1)/2$ possible pairings, 4% corresponded to the same CAT class ("true positives").

In the second CATH experiment ("CATH prefiltering experiment") we used a further reduced subset of the above set to compare our method with prefiltering stages in SMM and VAST. The set was reduced to the set for which all three methods (SSM, VAST and ours) were producing output, and for which a reasonable correspondence between the CATH definition of structural domain, and the one used in VAST (see below) could be established. Sources of failure in SSM and VAST were not investigated. This resulted in a test set consisting of 1528 structural domains, with 3% true positives.

Similarly, in the third CATH experiment ("CATH substructure experiment", subsection "Detecting a substructure in a set of larger structures"), we used a subset of CATH domains for which the three used methods (here SSM, SGM and our method) were producing output Sources of failure in SSM and SGM were not investigated. This resulted in a test set consisting of 652 structural domains, matched against the set of 652 full structures they were extracted from, with 1% true positives.

In "Dyndom experiment" (subsection "Finding conformationally related structures") each structure in the test set had at least one conformationally related partner, and some of them had two, resulting in 345 true positives out of $677(677-1)/2$ possible pairings (self matching excluded). Transformation descriptors[5, 6] for the transformations connecting the pairs of structures used here are the median angle of 18° (the second and the third quartile between 10° and 34°), median translation along the transformation axis of $0.4\AA$ (quartiles: $0.2\AA$ to $0.9\AA$), and median closure of 82% (56% to 96%).

Parametrization of the method presented in the text

The main parametrization choices the user has to make in the current implementation are the choice of the Gaussian width δ (Eq. 2, in the main text), whether to group similar directions in space (see "Implementation" subsection in the main text), and whether to enforce the length match (Eq. 7 in the main text) among SSEs. As illustrated in Fig. 2 in the case of "CATH experiment", the optimal values of δ lay somewhere in the range $[0.2, 0.5]$ and the improvements saturates very fast for $\delta > 0.3$. On the flip side, bigger values of δ leave more work to be done for the conjugate gradient search for the optimal R.

The times for the runs shown in Fig. 2 are given in Table 1. In experiments which grouped SSE's by direction, the criterion was that the cosine of the angle between the two be greater or equal to 0.98, and in length mismatch penalizing terms $tol_{\Delta L}$ from Eq. 7 in the main text was set to be 5 residues for strands, and 10 residues for helices.

During the development of argument, the question arose about the role of the length of SSEs in recognizing a related fold, such as in the "CATH experiment" described here. As it turns out, the scheme shows quite reasonable behavior: if the experiments requires classifying a fold correctly down to 4 CATH numbers (not only 3 as in the experiment in Fig 3, that we took literally from Kolodny et al. [4]), in that case imposing the length match does help, as shown in Fig. 3 here. In that figure we are showing the results of the experiment in which a set of 488 domains classified as 4 helix bundles (CATH 1.20.120) had to be correctly binned according to the 4th CATH number, the test set containing 7% true positive (TP) cases.

Another possibility we investigated was replacing the Needleman-Wunsch alignment algorithm with Smith-Waterman. It resulted in no detectable change in the performance (data not shown).

The choice of parameterization and algorithms depending on the intended application certainly merits further investigation.

Table 1: Runtimes for "CATH experiment" on a 3GHz CPU for different parametrization choices.

δ	SSEs grouped	ΔL penalty	time (min)
0.1	0	0	33
		1	29
	1	0	19
		1	19
0.2	0	0	54
		1	49
	1	0	28
		1	26
0.3	0	0	94
		1	84
	1	0	47
		1	42
0.4	0	0	161
		1	143
	1	0	85
		1	78
0.5	0	0	171
		1	149
	1	0	90
		1	79

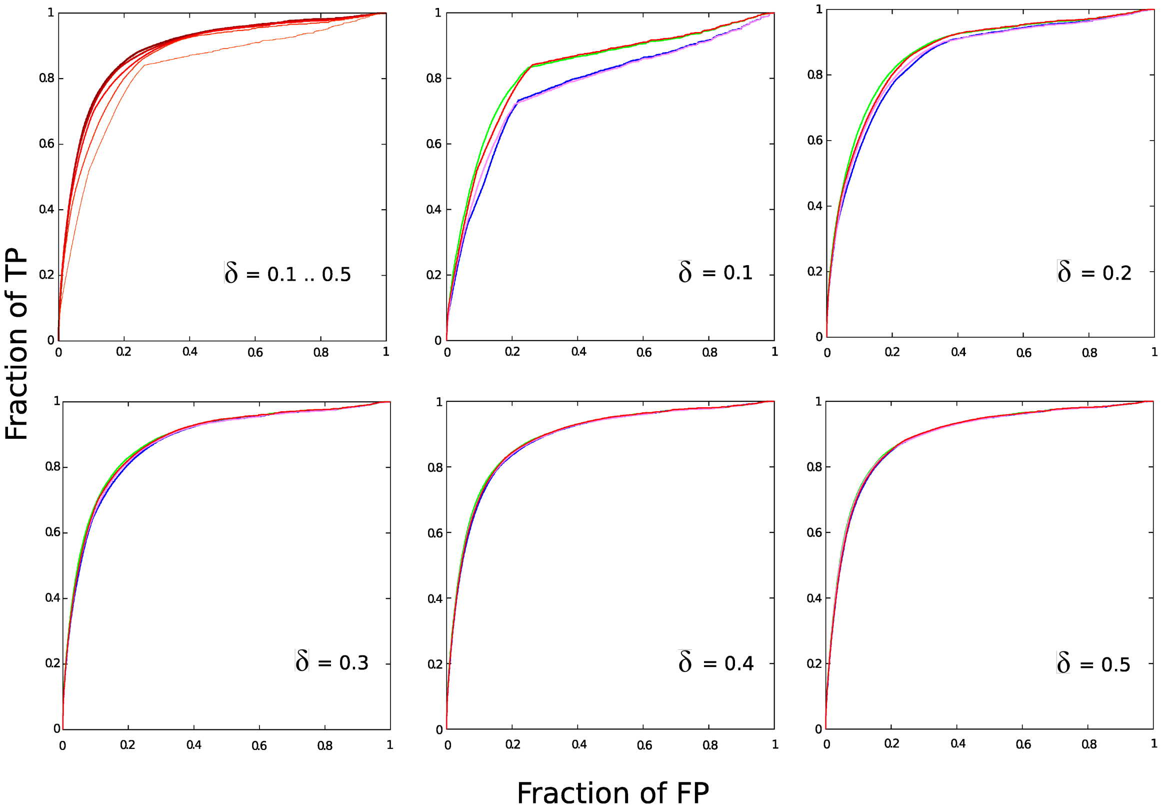

Figure 2: Performance as a function of parametrization. Top left panel overlays ROC curves for different choices of δ, without length mismatch penalty or grouping of SSEs according to direction, to show the saturation close after $\delta = 0.3$. In all panels the coloring scheme is the same. Red: no grouping and no length mismatch penalty. Green: no grouping, but with length mismatch penalty. Blue: SSEs grouped by direction, no length mismatch penalty. Pink: SSEs grouped by direction, length mismatch penalty on. The values of δ are indicated in each panel.

Parametrization of other methods

When not stated otherwise, the methods used in the paper were used with their default parametrization.

SSM. In runs using SSM[7] in its original implementation, as as a high resolution method, we used it in its pairwise matching mode, so no cutoff in similarity was imposed; information about the sequential order of SSEs was used, to make the search comparable to our implementation. In flexible matching experiment the precision was set to normal, and in substructure matching experiment to lowest, to increase the number of hits, because it seemed that SSM was not plagued with too many false positives there. The results were sorted using SSM's preferred scoring system (Q-score).

In "Classification of structural domains" we tried to extricate the pre-filtering stage of SSM, to compare it on something of an equal footing with the prefilter we have proposed. The immediate problem with this project was that SSM does not

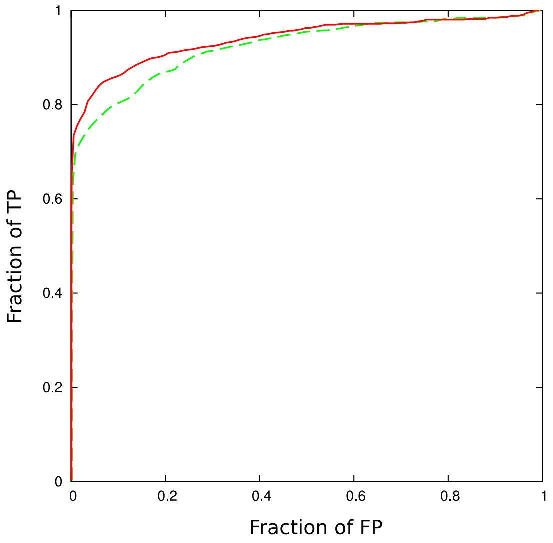

Figure 3: Improving the classification down to 4 CATH numbers by imposing the length match. Red, full line: with the length mismatch penalty, Eq.7 in the main text, using $tol_{\Delta L}$ set to 10 residues for a helix and 5 residues for a strand. Green, dashed: no length penalty.

assign anything like a continuous score in our sense, not at this stage of comparison. Since SSM uses five heuristic levels of resolution[7], our first line of attack consisted in the attempt to bin the results according to the highest level of precision resulting in *highest_p*[8] quantity (being apparently the highest fraction of protein structure assigned in the detected subgraphs) greater than 0.5. This resulted in the dashed line ROC in the inset of Fig. 3 in the main text. That performance clearly is not in the accordance with the amount of information that SSM is handling at that point, so we resorted to a machine learning approach. We binned *highest_p* quantity, that ranges between 0.0 and 1.0 into 10 bins, and assigned the following score to each pair of compared structures:

$$SSM = \sum_{l=1}^{6} \varepsilon_l(b) highest\_p_l. \tag{7}$$

Here the index l runs over all 5 levels of resolution used by SSM, plus the fictive 6th level, corresponding to the case of SSM reporting that no match was possible under any resolution criteria. The parameter b refers to one of the 10 bins that the values of *highest_p* were assigned to. The 51 values ε_{lb} were treated as parameters to be optimized, through a Metropolis Monte Carlo procedure, to obtain the largest possible area under the ROC curve. They were optimized on the subset consisting of 1% of all pairs used in the "CATH prefiltering experiment." The optimized parameters were then used to produce the ROC curve on the full set, shown as full orange line in the inset of Fig. 3 in the main text.

VAST. In all runs VAST [11] was used with its default parametrization, and its own definitions of structural domains. The pre-processed input domains for VAST were downloaded from ftp://ftp.ncbi.nih.gov/mmdb/vsq.files.

SGM. In all runs we used SGM [9] in its GIT[10] edition, with "smoothing backbone" option, using 30 Gauss integral based measures, plus the one based on the length of the smoothened curve. The similarity between structures was scored using Euclidean distance between the two points in 31-dimensional space [9]. Following the requirements of the method, structures longer than 874 residues and/or missing more than 3 C_α's in the middle of the structure were not considered.

Obtaining the methods from the text

The methods used in the text were obtained from their respective websites (see references in the main text), except for Sabertooth and 3Dhit that were obtained from the authors (M. Porto and D. Plewczynski, respectively). Our own implementation is available at http://epsf.bmad.bii.a-star.edu.sg.

The possible asymmetry in the initial guess

Another potentially troublesome point in our algorithm may conceivably arise in practice from the order in which the initial guesses for the optimizing rotation R are made (see the main text, "Implementation," first paragraph). Here we show results of an experiment in which this feature of the algorithm is not particularly harmful to the overall performance.

We repeat the "CATH substructure experiment" (see "Data sets," above) for three re-orderings of the quadruple loop - a generic one, the one in which the identities of X and Y (query and target) are switched, and the one in which the identities of the first an the second element of each pair are switched (note that this has to be done at the same time, to preserver the sequential ordering). As seen in Fig. 4, while there exists a difference in the ROC curve (indicating a possible direction of improvement of the algorithm) it does not seem big enough to call for an urgent overhaul of the algorithm.

References

[1] Karney, C (2007) Quaternions in molecular modeling. *J Mol Graph Model* 25:595

[2] Hobohm, U, Scharf, M, Schneider, R, Sander, C (1992) Selection of representative protein data sets. *Protein Science* 1:409

[3] Berman, H et al. (2000) The protein data bank. *Nucleic Acids Res* 28:235–242.

[4] Kolodny, R, Koehl, P, Levitt, M (2005) Comprehensive Evaluation of Protein Structure Alignment Methods: Scoring by Geometric Measures. *J Mol Biol* 346:1173–1188.

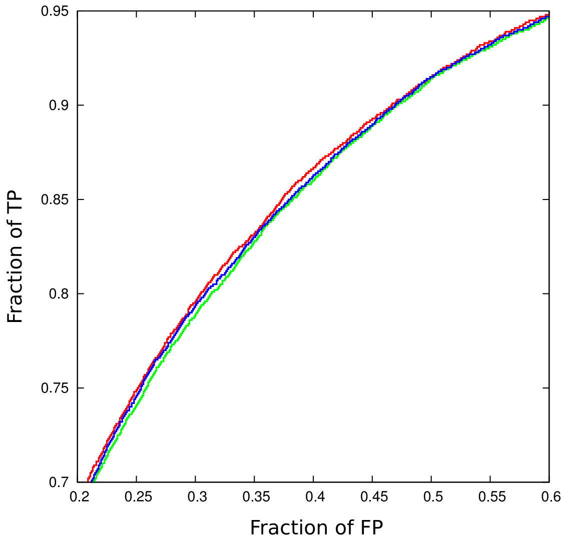

Figure 4:
The region (notice the axes range) of the ROC curve showing the largest dependence on the order of picking the initial guess in looking for the optimizing rotation. Different colors indicate three different strategies - see "The possible asymmetry in the initial guess" in this document.

[5] Hayward, S, Kitao, A, Berendsen, H (1997) Model-Free Methods of Analyzing Domain Motions in Proteins From Simulation: A Comparison of Normal Mode Analysis and Molecular Dynamics Simulation of Lysozyme. *Proteins* 27:425–437.

[6] Hayward, S, Berendsen, H (1998) Systematic Analysis of Domain Motions in Proteins From Conformational Change: New Results on Citrate Synthase and T4 Lysozyme. *Proteins* 30:144–154.

[7] Krissinel, E, Henrick, K (2004) Secondary-structure matching (SSM), a new tool for fast protein structure alignment in three dimensions. *Acta Crystallogr D Biol Crystallogr* 60:2256–2268.

[8] E. Krissinel (2002-2004). SSM implementation. Available as a part of CCP4 distribution (http://www.ccp4.ac.uk)

[9] Roegen, P, Fain, B (2003) Automatic classification of protein structure by using Gauss integrals. *Proc Natl Acad Sci U S A* 100:119–124.

[10] Roegen, P (2005) Evaluating protein structure descriptors and tuning Gauss integral based descriptors. *J Phys: Condens Matter* 17:S1523–S1538.

[11] Madej, T., Gibrat, J., and Bryant, S. (1995). Threading a database of protein cores. *Protein Struct. Funct. Genet*, **23**(3), 356–369.
